# Supplementary material for: Unveiling the Bioactive Potential of the Invasive Jellyfish Phyllorhiza punctata Through Integrative Transcriptomic and Proteomic Analyses
Source: Biomolecules. 2025 Aug 4;15(8):1121. doi: 10.3390/biom15081121 (PMC12383608; doi:10.3390/biom15081121)
Supplement: Supplementary file 1 [file biomolecules-15-01121-s001.zip › Suplementary Material/FileS3/PEPFOLD4/XP_065053736_2722/clusters.html]

 PEP-FOLD clusters


Download archive of all models  here   

|  |  |  |
| --- | --- | --- |
| # | file | sOPEP |
| # cluster 1 - Size: 1 |  |  |
|  | XP\_065053736\_2722-model1 | -13.9419 |
| # cluster 2 - Size: 3 |  |  |
|  | XP\_065053736\_2722-model2 | -12.952 |
|  | XP\_065053736\_2722-model2.1 | -7.78397 |
|  | XP\_065053736\_2722-model2.2 | -6.32243 |
| # cluster 3 - Size: 2 |  |  |
|  | XP\_065053736\_2722-model3 | -12.0859 |
|  | XP\_065053736\_2722-model3.1 | -6.44816 |
| # cluster 4 - Size: 2 |  |  |
|  | XP\_065053736\_2722-model4 | -11.4135 |
|  | XP\_065053736\_2722-model4.1 | -6.64864 |
| # cluster 5 - Size: 1 |  |  |
|  | XP\_065053736\_2722-model5 | -10.8279 |
| # cluster 6 - Size: 2 |  |  |
|  | XP\_065053736\_2722-model6 | -10.5705 |
|  | XP\_065053736\_2722-model6.1 | -5.85661 |
| # cluster 7 - Size: 2 |  |  |
|  | XP\_065053736\_2722-model7 | -10.4674 |
|  | XP\_065053736\_2722-model7.1 | -9.78353 |
| # cluster 8 - Size: 1 |  |  |
|  | XP\_065053736\_2722-model8 | -9.25446 |
| # cluster 9 - Size: 1 |  |  |
|  | XP\_065053736\_2722-model9 | -9.04117 |
| # cluster 10 - Size: 1 |  |  |
|  | XP\_065053736\_2722-model10 | -8.83258 |
| # cluster 11 - Size: 2 |  |  |
|  | XP\_065053736\_2722-model11 | -8.76414 |
|  | XP\_065053736\_2722-model11.1 | -8.28941 |
| # cluster 12 - Size: 3 |  |  |
|  | XP\_065053736\_2722-model12 | -8.29524 |
|  | XP\_065053736\_2722-model12.1 | -7.43837 |
|  | XP\_065053736\_2722-model12.2 | -6.71421 |
| # cluster 13 - Size: 1 |  |  |
|  | XP\_065053736\_2722-model13 | -8.14889 |
| # cluster 14 - Size: 3 |  |  |
|  | XP\_065053736\_2722-model14 | -8.14312 |
|  | XP\_065053736\_2722-model14.1 | -7.2976 |
|  | XP\_065053736\_2722-model14.2 | -6.46808 |
| # cluster 15 - Size: 1 |  |  |
|  | XP\_065053736\_2722-model15 | -8.12623 |
| # cluster 16 - Size: 5 |  |  |
|  | XP\_065053736\_2722-model16 | -8.0006 |
|  | XP\_065053736\_2722-model16.1 | -7.41766 |
|  | XP\_065053736\_2722-model16.2 | -7.34606 |
|  | XP\_065053736\_2722-model16.3 | -6.584 |
|  | XP\_065053736\_2722-model16.4 | -5.60915 |
| # cluster 17 - Size: 2 |  |  |
|  | XP\_065053736\_2722-model17 | -7.90265 |
|  | XP\_065053736\_2722-model17.1 | -5.31883 |
| # cluster 18 - Size: 1 |  |  |
|  | XP\_065053736\_2722-model18 | -7.61079 |
| # cluster 19 - Size: 6 |  |  |
|  | XP\_065053736\_2722-model19 | -7.4726 |
|  | XP\_065053736\_2722-model19.1 | -6.45221 |
|  | XP\_065053736\_2722-model19.2 | -6.09459 |
|  | XP\_065053736\_2722-model19.3 | -5.66199 |
|  | XP\_065053736\_2722-model19.4 | -5.35921 |
|  | XP\_065053736\_2722-model19.5 | -4.30782 |
| # cluster 20 - Size: 1 |  |  |
|  | XP\_065053736\_2722-model20 | -7.45931 |
| # cluster 21 - Size: 5 |  |  |
|  | XP\_065053736\_2722-model21 | -7.39597 |
|  | XP\_065053736\_2722-model21.1 | -6.98182 |
|  | XP\_065053736\_2722-model21.2 | -6.86356 |
|  | XP\_065053736\_2722-model21.3 | -5.80358 |
|  | XP\_065053736\_2722-model21.4 | -4.49465 |
| # cluster 22 - Size: 2 |  |  |
|  | XP\_065053736\_2722-model22 | -7.29753 |
|  | XP\_065053736\_2722-model22.1 | -6.2002 |
| # cluster 23 - Size: 1 |  |  |
|  | XP\_065053736\_2722-model23 | -7.24077 |
| # cluster 24 - Size: 1 |  |  |
|  | XP\_065053736\_2722-model24 | -7.1663 |
| # cluster 25 - Size: 1 |  |  |
|  | XP\_065053736\_2722-model25 | -7.10185 |
| # cluster 26 - Size: 1 |  |  |
|  | XP\_065053736\_2722-model26 | -6.8699 |
| # cluster 27 - Size: 1 |  |  |
|  | XP\_065053736\_2722-model27 | -6.86614 |
| # cluster 28 - Size: 1 |  |  |
|  | XP\_065053736\_2722-model28 | -6.85932 |
| # cluster 29 - Size: 1 |  |  |
|  | XP\_065053736\_2722-model29 | -6.82451 |
| # cluster 30 - Size: 3 |  |  |
|  | XP\_065053736\_2722-model30 | -6.72416 |
|  | XP\_065053736\_2722-model30.1 | -5.41679 |
|  | XP\_065053736\_2722-model30.2 | -4.78037 |
| # cluster 31 - Size: 1 |  |  |
|  | XP\_065053736\_2722-model31 | -6.71655 |
| # cluster 32 - Size: 2 |  |  |
|  | XP\_065053736\_2722-model32 | -6.68078 |
|  | XP\_065053736\_2722-model32.1 | -6.35281 |
| # cluster 33 - Size: 1 |  |  |
|  | XP\_065053736\_2722-model33 | -6.56949 |
| # cluster 34 - Size: 1 |  |  |
|  | XP\_065053736\_2722-model34 | -6.52727 |
| # cluster 35 - Size: 2 |  |  |
|  | XP\_065053736\_2722-model35 | -6.51789 |
|  | XP\_065053736\_2722-model35.1 | -5.81024 |
| # cluster 36 - Size: 2 |  |  |
|  | XP\_065053736\_2722-model36 | -6.29384 |
|  | XP\_065053736\_2722-model36.1 | -5.94893 |
| # cluster 37 - Size: 2 |  |  |
|  | XP\_065053736\_2722-model37 | -6.21782 |
|  | XP\_065053736\_2722-model37.1 | -5.46123 |
| # cluster 38 - Size: 1 |  |  |
|  | XP\_065053736\_2722-model38 | -6.1686 |
| # cluster 39 - Size: 2 |  |  |
|  | XP\_065053736\_2722-model39 | -5.99859 |
|  | XP\_065053736\_2722-model39.1 | -5.36791 |
| # cluster 40 - Size: 1 |  |  |
|  | XP\_065053736\_2722-model40 | -5.94864 |
| # cluster 41 - Size: 1 |  |  |
|  | XP\_065053736\_2722-model41 | -5.78955 |
| # cluster 42 - Size: 1 |  |  |
|  | XP\_065053736\_2722-model42 | -5.74343 |
| # cluster 43 - Size: 1 |  |  |
|  | XP\_065053736\_2722-model43 | -5.71773 |
| # cluster 44 - Size: 2 |  |  |
|  | XP\_065053736\_2722-model44 | -5.70362 |
|  | XP\_065053736\_2722-model44.1 | -5.42171 |
| # cluster 45 - Size: 3 |  |  |
|  | XP\_065053736\_2722-model45 | -5.70183 |
|  | XP\_065053736\_2722-model45.1 | -5.52952 |
|  | XP\_065053736\_2722-model45.2 | -4.91025 |
| # cluster 46 - Size: 1 |  |  |
|  | XP\_065053736\_2722-model46 | -5.49571 |
| # cluster 47 - Size: 2 |  |  |
|  | XP\_065053736\_2722-model47 | -5.26511 |
|  | XP\_065053736\_2722-model47.1 | -4.49282 |
| # cluster 48 - Size: 1 |  |  |
|  | XP\_065053736\_2722-model48 | -5.25715 |
| # cluster 49 - Size: 1 |  |  |
|  | XP\_065053736\_2722-model49 | -5.07489 |
| # cluster 50 - Size: 1 |  |  |
|  | XP\_065053736\_2722-model50 | -5.0599 |
| # cluster 51 - Size: 2 |  |  |
|  | XP\_065053736\_2722-model51 | -5.05173 |
|  | XP\_065053736\_2722-model51.1 | -5.00917 |
| # cluster 52 - Size: 1 |  |  |
|  | XP\_065053736\_2722-model52 | -4.9624 |
| # cluster 53 - Size: 3 |  |  |
|  | XP\_065053736\_2722-model53 | -4.73607 |
|  | XP\_065053736\_2722-model53.1 | -4.61967 |
|  | XP\_065053736\_2722-model53.2 | -4.47149 |
| # cluster 54 - Size: 4 |  |  |
|  | XP\_065053736\_2722-model54 | -4.51782 |
|  | XP\_065053736\_2722-model54.1 | -4.38173 |
|  | XP\_065053736\_2722-model54.2 | -4.33144 |
|  | XP\_065053736\_2722-model54.3 | -4.23024 |
| # cluster 55 - Size: 1 |  |  |
|  | XP\_065053736\_2722-model55 | -4.37921 |
| # cluster 56 - Size: 1 |  |  |
|  | XP\_065053736\_2722-model56 | -4.21486 |
| # cluster 57 - Size: 1 |  |  |
|  | XP\_065053736\_2722-model57 | -4.21295 |
|  |  |  |
